# Nerolidol Mitigates Colonic Inflammation: An Experimental Study Using both In Vivo and In Vitro Models

**DOI:** 10.3390/nu12072032

**Published:** 2020-07-08

**Authors:** Vishnu Raj, Balaji Venkataraman, Saeeda Almarzooqi, Sanjana Chandran, Shreesh K. Ojha, Samir Attoub, Thomas E. Adrian, Sandeep B. Subramanya

**Affiliations:** 1 Department of Physiology, College of Medicine and Health Sciences, United Arab Emirates University, Al Ain 17666, UAE; rajvishnu@uaeu.ac.ae (V.R.); balajiv@uaeu.ac.ae (B.V.); sanjanachandran25@gmail.com (S.C.); 2 Zayed Bin Sultan Center for Health Sciences, College of Medicine and Health Sciences, United Arab Emirates University, Al Ain 17666, UAE; 3 Department of Pathology, College of Medicine and Health Sciences, United Arab Emirates University, Al Ain 17666, UAE; saeeda.almarzooqi@uaeu.ac.ae; 4 Department of Pharmacology and Therapeutics, College of Medicine and Health Sciences, United Arab Emirates University, Al Ain 17666, UAE; shreeshojha@uaeu.ac.ae (S.K.O.); samir.attoub@uaeu.ac.ae (S.A.); 5 Department of Basic Medical Sciences, Mohamed Bin Rashid University of Medicine and Health Sciences, Dubai 505055, UAE; Thomas.Adrian@mbru.ac.ae

**Keywords:** nerolidol, phytochemical, inflammatory bowel disease, bioactive compounds, inflammatory molecular mechanisms, colitis

## Abstract

Nerolidol (NED) is a naturally occurring sesquiterpene alcohol present in various plants with potent anti-inflammatory effects. In the current study, we investigated NED as a putative anti-inflammatory compound in an experimental model of colonic inflammation. C57BL/6J male black mice (C57BL/6J) were administered 3% dextran sodium sulfate (DSS) in drinking water for 7 days to induce colitis. Six groups received either vehicle alone or DSS alone or DSS with oral NED (50, 100, and 150 mg/kg body weight/day by oral gavage) or DSS with sulfasalazine. Disease activity index (DAI), colonic histology, and biochemical parameters were measured. TNF-α-treated HT-29 cells were used as in vitro model of colonic inflammation to study NED (25 µM and 50 µM). NED significantly decreased the DAI and reduced the inflammation-associated changes in colon length as well as macroscopic and microscopic architecture of the colon. Changes in tissue Myeloperoxidase (MPO) concentrations, neutrophil and macrophage mRNA expression (CXCL2 and CCL2), and proinflammatory cytokine content (IL-1β, IL-6, and TNF-α) both at the protein and mRNA level were significantly reduced by NED. The increase in content of the proinflammatory enzymes, COX-2 and iNOS induced by DSS were also significantly inhibited by NED along with tissue nitrate levels. NED promoted Nrf2 nuclear translocation dose dependently. NED significantly increased antioxidant enzymes activity (Superoxide dismutase (SOD) and Catalase (CAT)), Hemeoxygenase-1 (HO-1), and SOD3 mRNA levels. NED treatment in TNF-α-challenged HT-29 cells significantly decreased proinflammatory chemokines (CXCL1, IL-8, CCL2) and COX-2 mRNA levels. NED supplementation attenuates colon inflammation through its potent antioxidant and anti-inflammatory activity both in in vivo and in vitro models of colonic inflammation.

## 1. Introduction

Inflammatory bowel diseases (IBD) is an umbrella term that comprises the chronic inflammatory conditions of gastrointestinal tract, which are categorized as Crohn’s disease (CD) and ulcerative colitis (UC) [1,2].

Though the exact mechanisms underlying the pathophysiology of UC are not clearly understood, it is a multifactorial disease where an interplay between environmental factors and genetic susceptibility, together with a dysregulated immune response, could be the major cause for the onset of this inflammatory condition [3,4]. CD is categorized as inappropriate immune response of Th1 pathway and involvement of various mechanisms in different periods of disease progression [1]. UC is associated with impairment of gut barrier integrity [5], inflammatory and immune responses in the mucosa, and elevated oxidative stress [6,7,8,9,10]. Activation of macrophages with an elevated production of proinflammatory cytokines such as tumor necrosis factor α (TNF-α), interferon-γ (IFN-γ), interleukin (IL)-1β, IL-6, and IL-12 are important features of UC [11,12]. The current pharmacological therapy for UC includes sulfasalazine, corticosteroids, immunosuppressive agents such as azathioprine, and biological therapy represented by the anti-TNF-α antibody to suppress the aberrant immune responses and inflammatory reactions [13]. However, the adverse effects associated with these prescription drugs over prolonged treatment periods, and the high relapse rates, limits their use [11,14]. Moreover, it has been reported that 40 % of the patients with IBD use some form of herbal or dietary supplements in addition to their medications [15]. For centuries, medicinal plants have been an essential source for many pharmacologically active agents, and still, a large number of the present medications are plant-derived products or their subsidiaries [16,17,18,19].

Nerolidol (NED) [3,7,11-trimethyl-1,6,10-dodecatrien-3-ol] is an aliphatic sesquiterpene, commonly found in essential oils from plants with floral odor. High content of (*E*)-NED is found in Oolong tea (a traditional semi-oxidized Chinese tea) and is known to contribute to the floral aroma to tea (*Camellia sinensis*) [20]. Madeira wines from Portugal are known to contain NED [21] it also contributes to the flavor of kiwifruit [22] and the aroma of strawberries [23]. The US Food and Drug Administration (FDA) has categorized NED as “generally regarded as safe” (GRAS) and has approved its use as a flavor enhancer in food industry [24]. Scientific reports provided evidence for its therapeutic efficacy in neurodegenerative diseases. It also possesses anti-microbial, anti-biofilm, antioxidant, anti-parasitic, skin-penetration enhancer, skin-repellent, anti-nociceptive, anti-inflammatory, and anti-cancer properties [24,25]. A NED derivative prevents oxidative injury in human lung epithelial cells via the activation of nuclear factor erythroid 2-related factor 2 (Nrf2). The cyto-protective transcription factor Nrf-2 mediates the cellular antioxidant defense mechanism. [26]. Several recent studies have shown that NED has anti-inflammatory effects [24,27,28]. In the current study, we evaluated the effect of NED in colonic inflammation using both in vivo and in vitro models.

## 2. Materials and Methods

### 2.1. Chemicals and Reagents

Dextran Sulfate Sodium—DSS (Molecular weight 36,000–50,000 Da) and NED were purchased from Sigma-Aldrich, (St. Louis, MO, USA). MPO, IL-6, IL-1β, and TNF-α enzyme-linked immunosorbent assay (ELISA) kits were purchased from R&D systems (Minneapolis, MN, USA). The reverse transcription kit and SYBR select mastermix were purchased from Applied Biosystems (Foster City, CA, USA). Macrogen Inc. (Seoul, South Korea), supplied primers used in qPCR. Protease and phosphatase inhibitor cocktail tablets were purchased from Roche (Basel, Switzerland). Antibodies were purchased from Abcam (Cambridge, MN, USA). The Pierce^TM^ BCA protein estimation kit, PVDF membrane, TRIZOL reagent, cytoplasmic and nuclear fraction isolation NE-PER nuclear extraction kit, and West Pico super signal chemiluminescent substrate were purchased from Thermo-Scientific (Waltham, MA, USA). RIPA buffer was purchased from Millipore (St. Louis, MO, USA). Dulbecco’s modified Eagle’s medium was purchased from Pierce Hyclone (Fremont, CA, USA). Heat-inactivated fetal bovine serum was purchased from Gibco (Carlsbad, CA, USA). Cell Titer-Glo^®^ Luminescent Cell Viability Assay Kit was purchased from Promega (Madison, WI, USA).

### 2.2. Animals

Twelve-week-old male C57BL/6J mice weighing 25–30 g were obtained from the central animal facility, College of Medicine and Health Sciences, UAE University. The animals were housed in groups of 6 per cage at a temperature of 23 ± 1 °C, a 12-h light–dark cycle, and a humidity of 50–60% for 1 week before the experiment. Food and water were provided *ad libitum*. All studies were approved by the Institutional Animal Ethics Committee of UAE University (approval # ERA_2017_5599).

### 2.3. Experimental Design

Mice were randomly allocated to 6 groups (10 animals per group). Untreated control, DSS alone, DSS + NED in three different doses (50, 100 and 150 mg/kg body weight/day by oral gavage). The 6th group received Sulfasalazine (SAZ) as the standard treatment at a dose of 50 mg/kg body weight/day by oral gavage in DSS treated animals. DSS (3%) was prepared with drinking water freshly every day and the animals were treated for seven days. At the end of the treatment (with NED and SAZ) period, mice were euthanized using pentobarbital overdose (100 mg/kg body weight). After laparotomy, the colon was excised and measured using graduated scale along with caecum. The colon was then flushed with ice cold saline to remove the fecal content and then cut into 1 cm pieces, which were immediately snap frozen using liquid nitrogen and then transferred to a −80 °C freezer. These tissues were used later for protein and RNA analysis. For histopathological scoring, the colon pieces from the junction of the cecum were fixed using 10% formalin for histopathology.

### 2.4. Evaluation of the Clinical Score for Colitis and Construction of Disease Activity Index (DAI)

Mice were weighed daily, and the occurrence of diarrhea and bleeding was recorded. Scores were constructed based on parameters highlighted in Table 1 [29]. The scores for weight loss, diarrhea, and bleeding were used to compile the disease activity index (DAI).

### 2.5. Quantification of Proinflammatory Cytokines by Enzyme-Linked Immunosorbent Assay (ELISA)

The concentrations of myeloperoxidase (MPO), TNF-α, IL-1β, and IL-6 in the colon homogenate of the mice were measured using ELISA kits according to the manufacturer’s instructions.

### 2.6. Histopathological Evaluation

After saline wash, the proximal segment of each colon was placed in a tissue cassette and immersed in 10% formaldehyde overnight. Tissues were then dehydrated by increasing concentrations of ethanol and embedded in paraffin. Slices 2 μm thick from the paraffin sections were stained with hematoxylin and eosin for the histological evaluation. Histologic scoring for each sample was made by a pathologist who was blinded to the experimental protocol. Samples were graded for colonic inflammation and crypt damage based on the scoring system highlighted in Table 2 and Table 3, respectively.

### 2.7. RNA Extraction and Real Time RT-PCR

Total RNA was extracted from colon using the TRIZOL reagent, and cDNA conversion was carried out by using the High-Capacity cDNA Reverse Transcription kit. Real-time polymerase chain reaction (PCR) was performed using the Quant Studio 7 Flex Real-Time PCR System (Thermo Fisher Scientific, MD, USA) with SYBR Select Master Mix. The obtained data were normalized using 18 s RNA as a reference gene, and the comparative CT (2-ΔΔCT) method was used as relative quantification for mRNA expression [30]. Primer sequences for each gene, PMID, and gene accession number are provided in Table 4.

### 2.8. Preparation of Cytosolic and Nuclear Fractions

For the investigation of Nrf2 and Lamin B1 nuclear translocation by Western blot analysis, cytosolic and nuclear proteins were extracted. To separate cytoplasmic and nuclear fractions, the NE-PER nuclear extraction kit was used following the manufacturer’s instructions. Nuclear and cytoplasmic fractions were stored at −80 °C until use. The protein concentration of nuclear and cytosolic fractions was measured using BCA protein assay reagent.

### 2.9. Measurement of Superoxide Dismutase, Catalase Enzyme Activity, and Tissue Nitrite Concentration

Superoxide dismutase (SOD) was assayed by using the method of Kakkar et al., [31] based on 50% inhibition of the formation of NADH–phenazine methosulphate–nitroblue tetrazolium (NBT) formazan at 520 *nm*. One unit of the enzyme taken as the amount of enzyme required for 50% inhibition of NBT reduction/min/mg protein. Catalase activity (CAT) was determined by the method of Sinha [32]. The values of CAT activity were expressed as moles of H_2_O_2_ utilized/min/mg protein. The levels of nitric oxide (NO) in colon homogenate were measured using a Griess reagent by the method of Lu et al. [33]; nitrite concentration, an indicator of NO production, was calculated from a NaNO_2_ standard curve and expressed as µmoles/mg protein. Snap frozen colon samples processed for biochemical estimation on the following day of sample collection.

### 2.10. Western Blot

Frozen colonic tissue samples were thawed, transferred to bead tubes from Biospec (Bartlesville, OK, USA), and homogenized in RIPA buffer supplemented with a protease and phosphatase inhibitor cocktail tablets using the Precellys homogenizer (Bertin instruments, Bretonneux, France) (three times for 15 s at 6500× *g* for 5 cycles). Then, the homogenates underwent brief sonication. Protein fractions were separated by sodium dodecyl sulphate-polyacrylamide gel electrophoresis (SDS-PAGE) gels, transferred to polyvinylidene difluoride PVDF membrane, and immune blotted for COX-2, iNOS, KEAP, and Nrf2 using specific antibodies. Blots were normalized to GAPDH for the cytoplasmic proteins, and Lamin-1 was used as an internal control for Nrf-2 expression in the nuclear fraction.

### 2.11. HT-29 Cell Culture

Human colorectal cancer cells (HT-29) cells were obtained from ATCC (Manassas, VA, USA). The cells were maintained under standard cell culture conditions at 37 °C and 5% CO_2_ in a humidified incubator using high-glucose Dulbecco’s modified Eagle’s medium, supplemented with 100 U/mL penicillin, 100 µg/mL streptomycin, and 10% (*v/v*) heat-inactivated fetal bovine serum. Before reaching confluence, cells were subcultured. Cells were seeded on 6-well plates 24 h before treatment with 1.5 × 10^5^ cells per well. To induce inflammatory conditions, cells were incubated with 1 ng/mL TNF-α with or without NED (25 and 50 μM, NED, MW 222.37 g/mol). After another 24 h, cells were harvested for inflammatory marker genes (CXCL1, CXCL2, and IL-8) and COX-2 gene expression analysis [34].

### 2.12. Cell Viability Assay

Cells were seeded onto 96 well plates at 5000 cells per well and were incubated with various concentrations of NED (0, 12.5, 50, 100, 150, and 200 µM) for 24 h. At the end of the incubation period, cell viability was determined using Cell Titer-Glo^®^ Luminescent Cell Viability Assay (Promega Corporation, Madison, WI, USA), according to the manufacturer’s protocol. This estimates the amount of ATP used as a correlation for the number of metabolically viable cells in culture. The luminescent signal was measured using the GLOMAX Luminometer system. The data are presented as percent cell viability of experimental groups compared to that of control untreated cells.

### 2.13. Statistical Analysis

All statistical analysis was carried out using SPSS (Version 25, UAE University licensed) Software (Armonk, NY, USA). Comparisons between groups were performed by one-way analysis of variance (ANOVA), followed by Tukey’s post hoc test for multiple comparisons. Data are plotted as mean ± SEM in the figures. *p* values ˂ 0.05 were considered statistically significant.

## 3. Results

### 3.1. Effect of NED on Disease Activity Index (DAI), Colon Length, MPO Concentration, and CXCL2 and CCL2 mRNA

**Figure 1:**
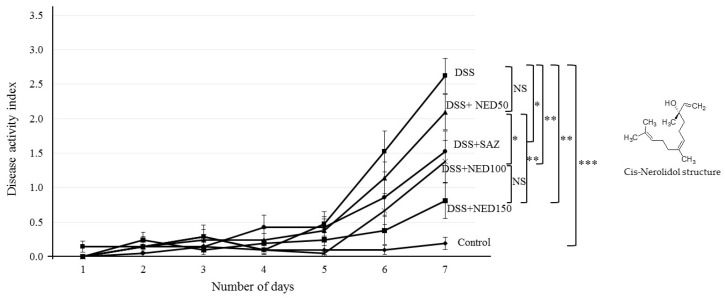
Effect of Nerolidol (NED) on disease activity index (DAI). Dextran sodium sulfate (DSS) treatment significantly increased the DAI score. Sulfasalazine (SAZ) and the higher concentrations of NED significantly prevented the increase in DAI scores induced by DSS. However, the lowest dose of NED (50 mg/kg) treatment had no effect. Data were obtained from *n* = 10 animals in each group and are expressed as mean ± SEM. *** *p* ≤ 0.001. ** *p* ≤ 0.01. * *p* ≤ 0.05. *P* values were obtained by one-way ANOVA followed by Tukey’s multiple comparison test using SPSS software, and *p* ≤ 0.05 was considered statistically significant.

In C5BL/6J mice, 3% DSS in drinking water induced distinct features of ulcerative colitis. The DAI score was high in the DSS treated group compared to the control group (*p* < 0.001). NED treatment of DSS-colitis animals at the higher doses (100 and 150 mg/kg bd wt) significantly (*p <* 0.01) improved the DAI scores. However, at the lowest dose of 50 mg/kg bd wt, NED treatment had no significant effect. SAZ treatment, as a positive control, showed significant (*p* < 0.05) improvement in DAI score (Figure 1).

**Figure 2:**
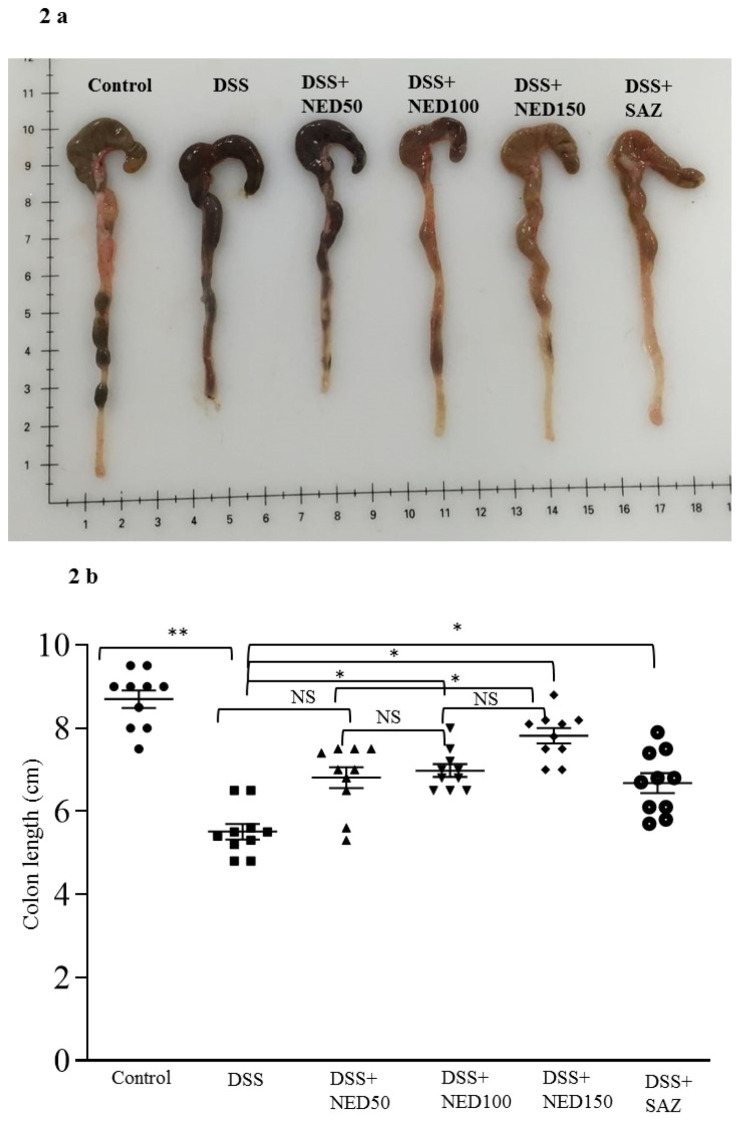

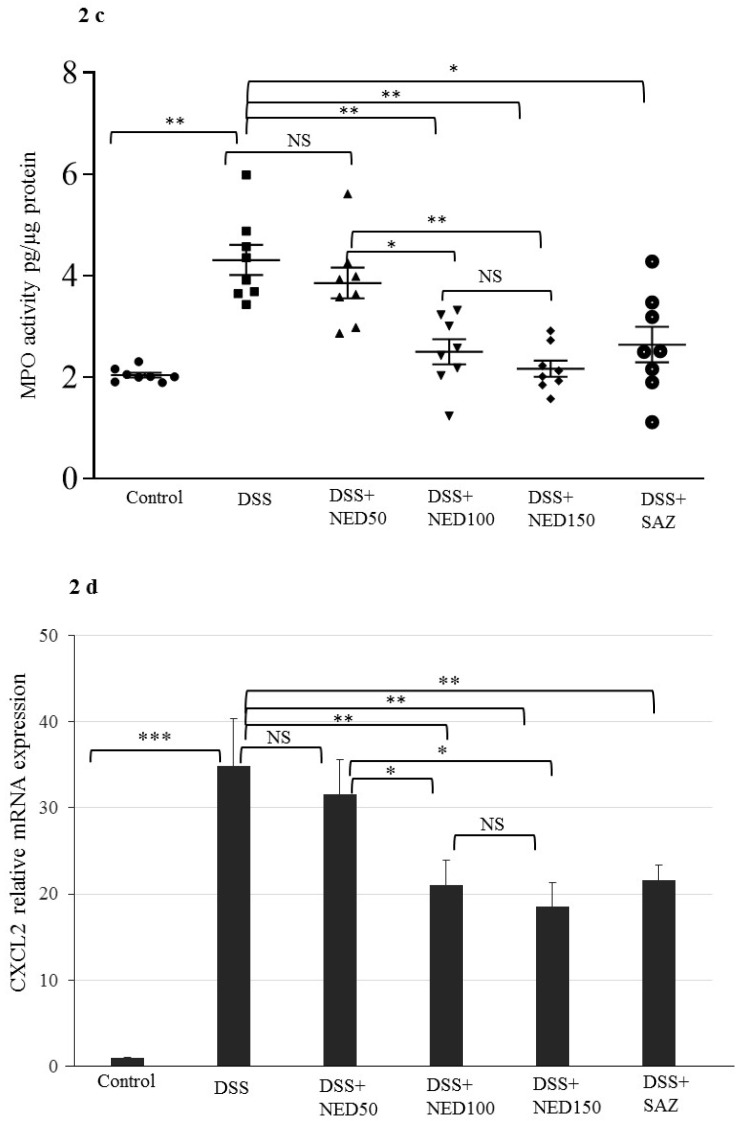

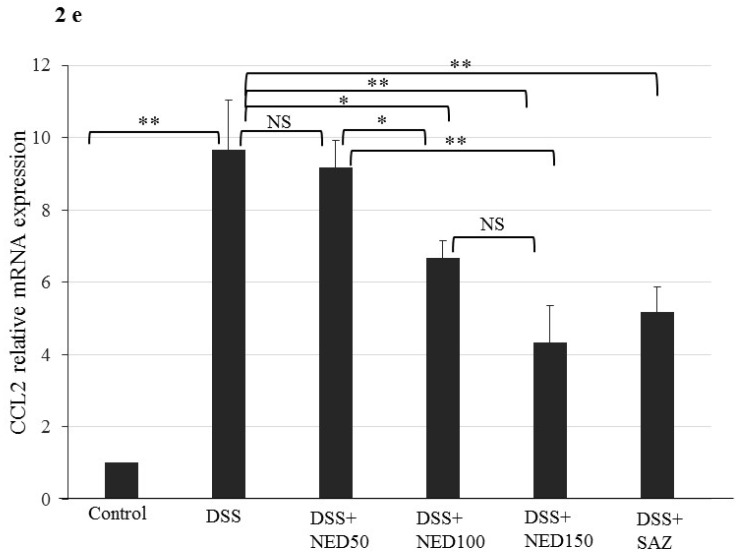
Effect of NED on colon length, MPO concentration, CXCL2 and CCL2 mRNA macrophage markers. The mean colon length was significantly decreased in the DSS-treated group. The higher doses of NED-treatment and SAZ-treatment significantly prevented the decrease in colon length compared to DSS group. The lowest concentration of NED had no effect (**a**,**b**). Data were obtained from *n* = 10 animals in each group and are expressed as means ± SEM. * *p* ≤ 0.05. DSS-treatment significantly increased MPO concentrations compared to untreated controls. The higher doses of NED-and SAZ reduced MPO concentrations compared to DSS treatment alone. However, the lowest dose of NED had no effect (**c**). Data were obtained from *n* = 10 animals in each group and are expressed as means ± SEM. ** *p* ≤ 0.01 * *p* ≤ 0.05. DSS-treatment significantly increased colonic CXCL2 and CCL2 mRNA levels compared to controls. The higher concentrations of NED and SAZ-treatment prevented the increase of both CXCL2 and CCL2 mRNA concentrations compared to DSS alone. However, the lowest concentration of NED-had no effect (**d**,**e**). Data were obtained from *n* = 8 animals in each group and are expressed as means ± SEM: *** *p* ≤ 0.001 ** *p* ≤ 0.01, and * *p* ≤ 0.05 were considered statistically significant, and NS indicates not significant.

DSS treatment-induced inflammation resulted in significantly (*p* < 0.01) decreased colon length. SAZ and NED treatment at the two higher doses (100 mg and 150 mg/kg) significantly prevented shortening of colon length (*p* < 0.05, Figure 2a,b). However, the lowest dose of NED was not effective in preventing the shortening of colon length due to DSS treatment. In the preliminary experiments conducted, NED treatment alone did not affect the colon length (data not shown).

MPO is an enzyme secreted from activated neutrophils and is commonly used as a marker to assess the level of neutrophil infiltration in the submucosa, indicating the degree of inflammation. DSS treatment significantly (*p* < 0.01) increased MPO concentrations compared to the untreated control group (Figure 2c). NED treatment at the two higher doses and SAZ treatment significantly decreased MPO concentration (*p* < 0.01, Figure 2c). However, the lowest dose (50 mg/kg) of NED had no significant effect

We measured mRNA expression of the chemokines CXCL2 and CCL2 as markers of macrophage infiltration. DSS treatment significantly increased the expression of both CXCL2 (*p* < 0.01) and CXCL2 (*p* < 0.001), indicating macrophage infiltration (Figure 2d,e). NED treatment at the two higher doses (100 and 150 mg/kg) and SAZ treatment significantly decreased the expression of both CXCL2 and CCL2 (Figure 2d,e). However, the lowest dose (50 mg/kg) of NED did not affect CXCL2 and CCL2 expression.

### 3.2. Effect of NED on Colon Microscopic Architecture

The control healthy group colon sections depicted typical architecture with normal thickness of the submucosa and muscle layers, as well as well-formed crypt structure in the mucosa. In the DSS-treated group, colonic inflammation penetrated the submucosa with focal loss of crypt and the surface epithelium. In contrast, the two higher dose NED-treated, DSS-induced inflammation groups, showed intact epithelium with minimum loss of crypt and inflammation. SAZ-treated DSS group also showed protection of colon microarchitecture. NED treatment alone did affect the microarchitecture of the colon (data not shown).

**Figure 3:**
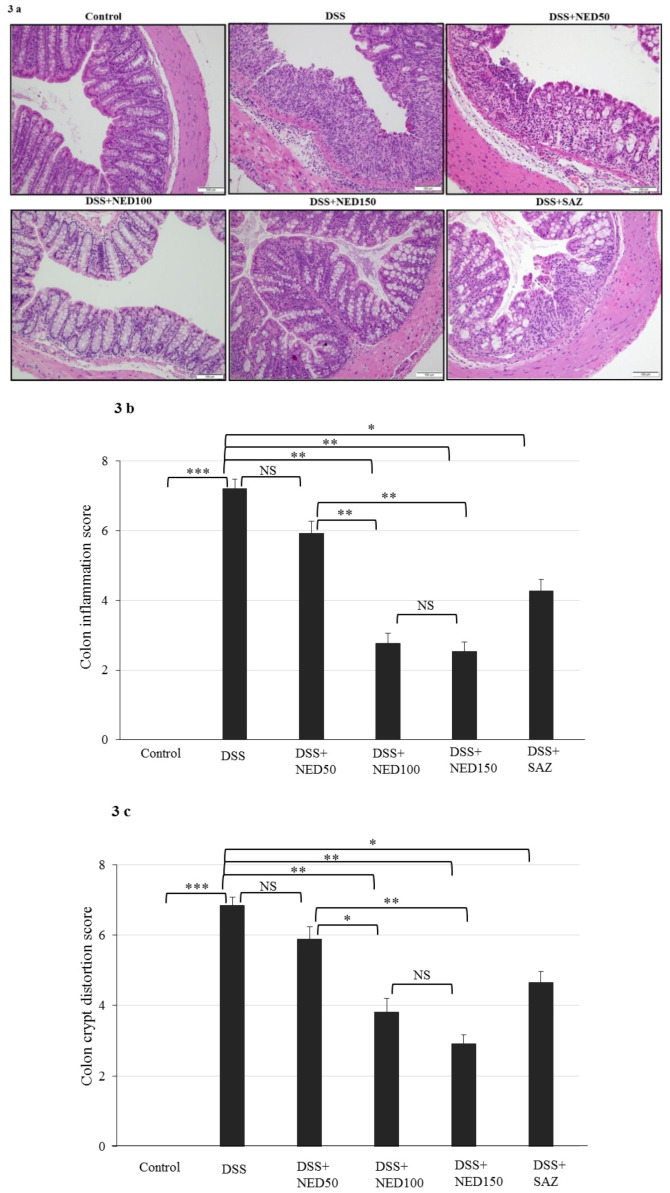
Effect of NED on colon histology. (**a**) Microscopic analysis shows typical architecture of the colon with normal thickness of the submucosa, muscle layer and regular crypt and villi structure in the mucosa in the control samples (Scale Bars: 100 µM). The DSS-induced colitis colon section shows focal loss of crypts and surface epithelium with inflammation reaching up to the submucosa. NED-treatment, at 100 and 150 mg/kg, protected the microscopic architecture in DSS-induced colitis. Crypt distortion score and colon inflammation scores were high in DSS-induced colitis group. NED treatment at 100 and 150 mg/kg significantly reduced both colon inflammation scores (**b**) and crypt distortion (**c**). The lowest concentration of NED (50 mg/kg) had no effect. Data were obtained from *n* = 8 animals in each group and are expressed as means ± SEM. *** *p* ≤ 0.001. ** *p* ≤ 0.01. * *p* ≤ 0.05. *p* values were obtained by one-way ANOVA followed by Tukey’s multiple comparison test using SPSS software. *p* ≤ 0.05 was considered statistically significant, and NS indicates not significant.

The most notable histological finding in the DSS-treated group was excessive crypt damage with edema and complete destruction of the colon surface epithelium (Figure 3a). The parameters used for scoring the colonic inflammation and crypt damage are highlighted in Table 2 and Table 3. As expected, the colonic inflammation score was statistically (*p* < 0.001) high in the DSS-treated group compared to the untreated control group (Figure 3b). NED treatment in DSS-induced inflammation significantly (*p* < 0.01) reduced the colonic inflammation score compared to DSS-treatment alone at the two higher doses (Figure 3b). As expected, SAZ treatment also reduced DSS-induced inflammation. However, the lowest dose of NED did not reduce inflammation. The crypt damage score was s higher in the DSS-treated group compared to the control group (*p* < 0.001, Figure 3c). NED-treatment protected crypt damage at the two higher doses (*p* < 0.01 Figure 3c). Significant protection of crypt damage was also observed in the SAZ-treated DSS group (*p* < 0.05). However, the lowest dose (50 mg/kg) of NED treatment did not provide any protection from crypt damage.

### 3.3. Effect of NED on Proinflammatory Cytokines and Proinflammatory Mediators

**Figure 4:**
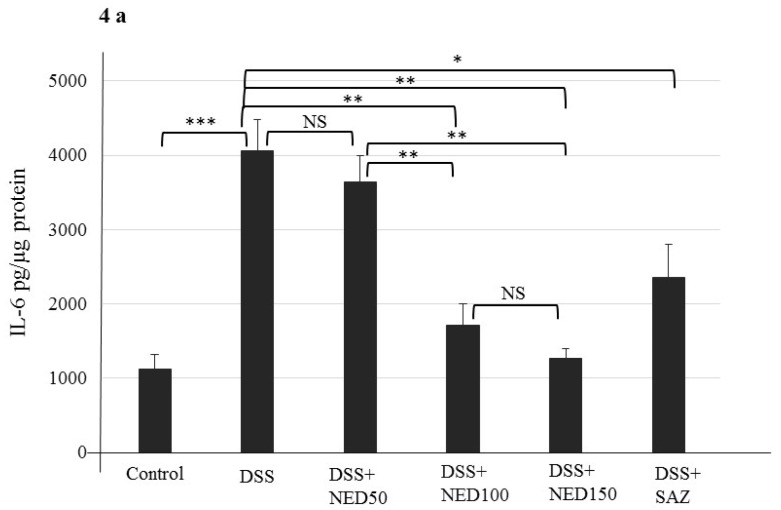

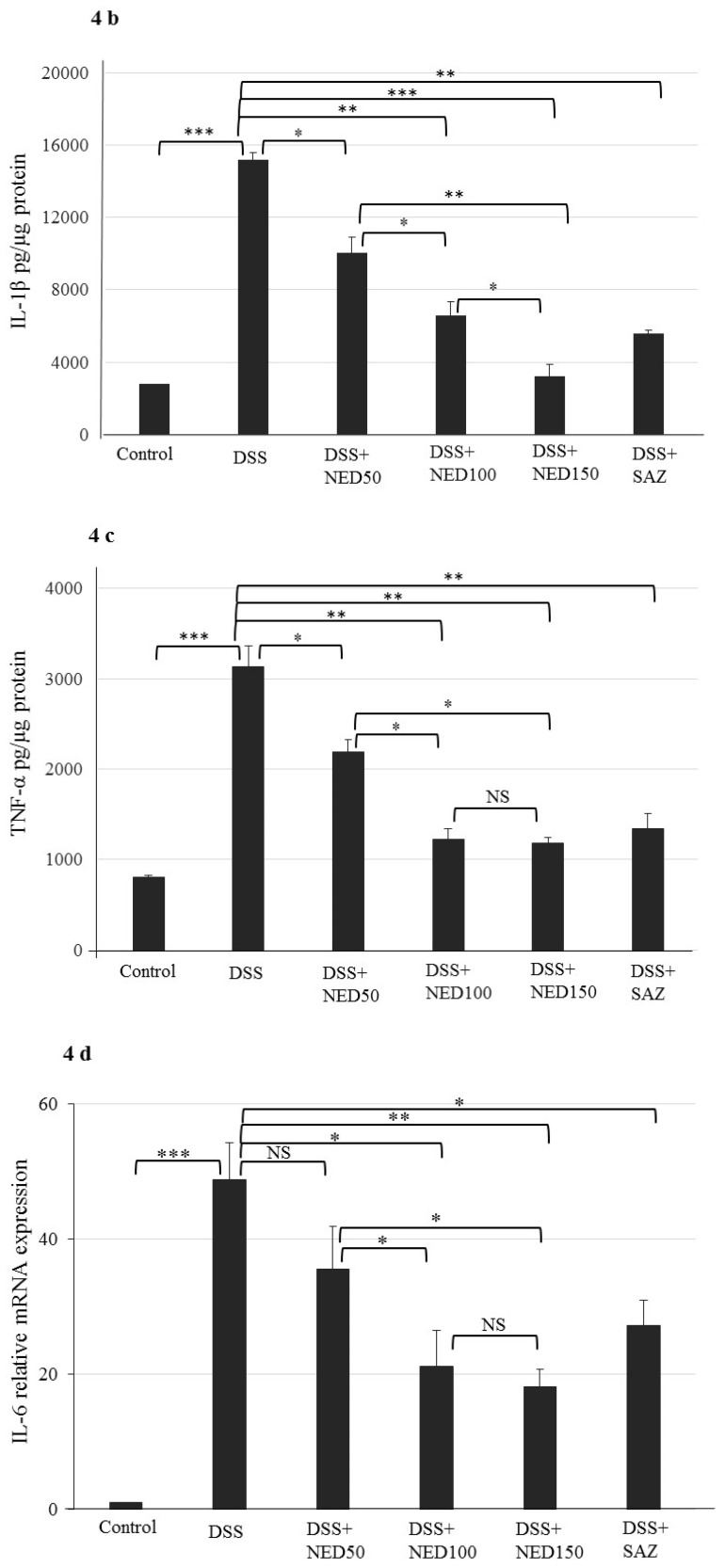

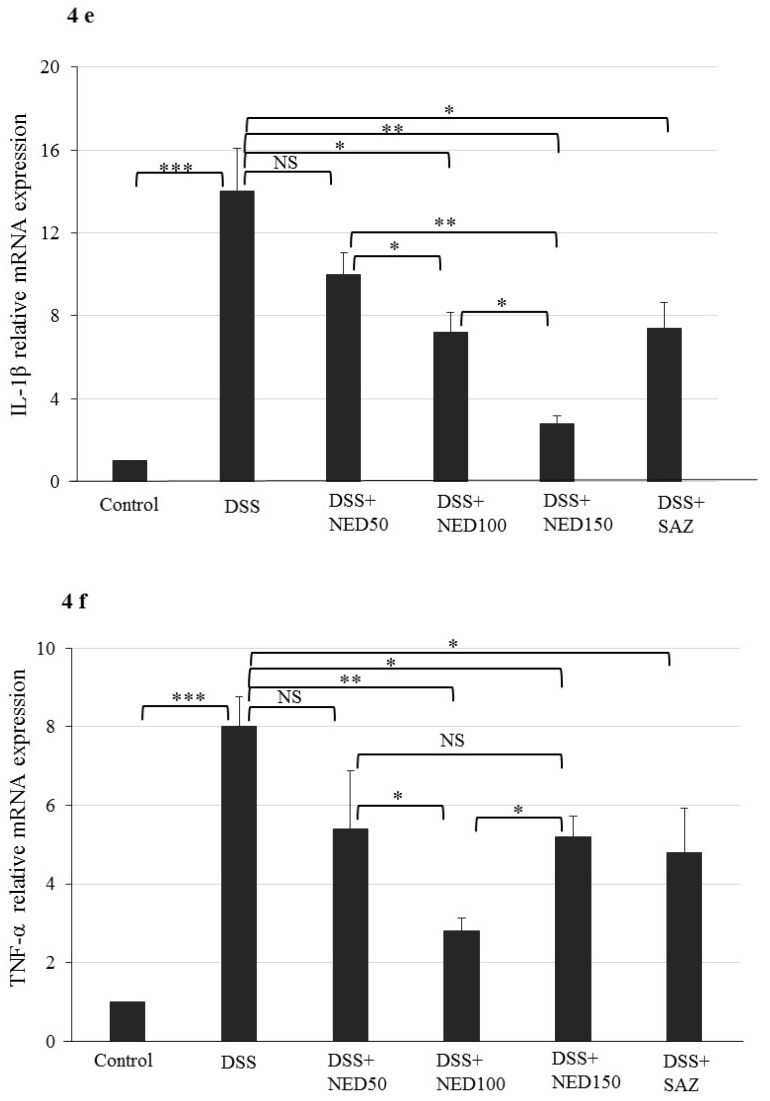
Effect of NED on proinflammatory cytokine protein and mRNA expression. NED-treatment, at 100 and 150 mg/kg, significantly inhibited the DSS-induced increase in concentrations of proinflammatory cytokines, at both the protein level (IL-6 (**a**), IL-1β (**b**), and TNF-α (**c**)) and mRNA expression levels (IL-6 (**d**), IL-1β (**e**), and TNF-α (**f**)) in DSS-induced colitis group. Data were obtained from *n* = 8 animals for ELISA and *n* = 6 animals for mRNA expression studies in each group and expressed as means ± SEM. *** *p* ≤ 0.001. ** *p* ≤ 0.01. * *p* ≤ 0.05. *p* values were obtained by one-way ANOVA followed by Tukey’s multiple comparison test using SPSS software. *p* ≤ 0.05 is considered statistically significant, and NS indicates not significant.

DSS treatment caused increases in concentrations of the proinflammatory cytokines statiscally: IL-6, IL-1β, and TNF-α, (all *p* < 0.001, Figure 4a–c). Concomitant increases in the relative expression of the mRNA for these respective cytokines (*p* < 0.001) were seen compared to the untreated control group (Figure 4d–f). NED-treated animals had significantly lower concentrations of these cytokines and their respective mRNAs than the DSS-treated control group (*p* < 0.01 for IL-6 and IL-1β at 100 mg and 150 mg/kg NED, *p* < 0.01 at 100 mg and *p* < 0.001 at 150 mg/kg for TNF-α) (Figure 4). However, the lowest dose of NED did not provide any protection from proinflammatory cytokine expression or release. SAZ treatment also reduced (*p* < 0.05) the proinflammatory cytokines expression and release (Figure 4). Since these results indicate that the lowest dose of NED (50 mg/kg) is not effective in limiting DSS-induced inflammatory responses, this group was omitted from subsequent studies. Similarly, the SAZ group was also removed from further studies.

**Figure 5:**
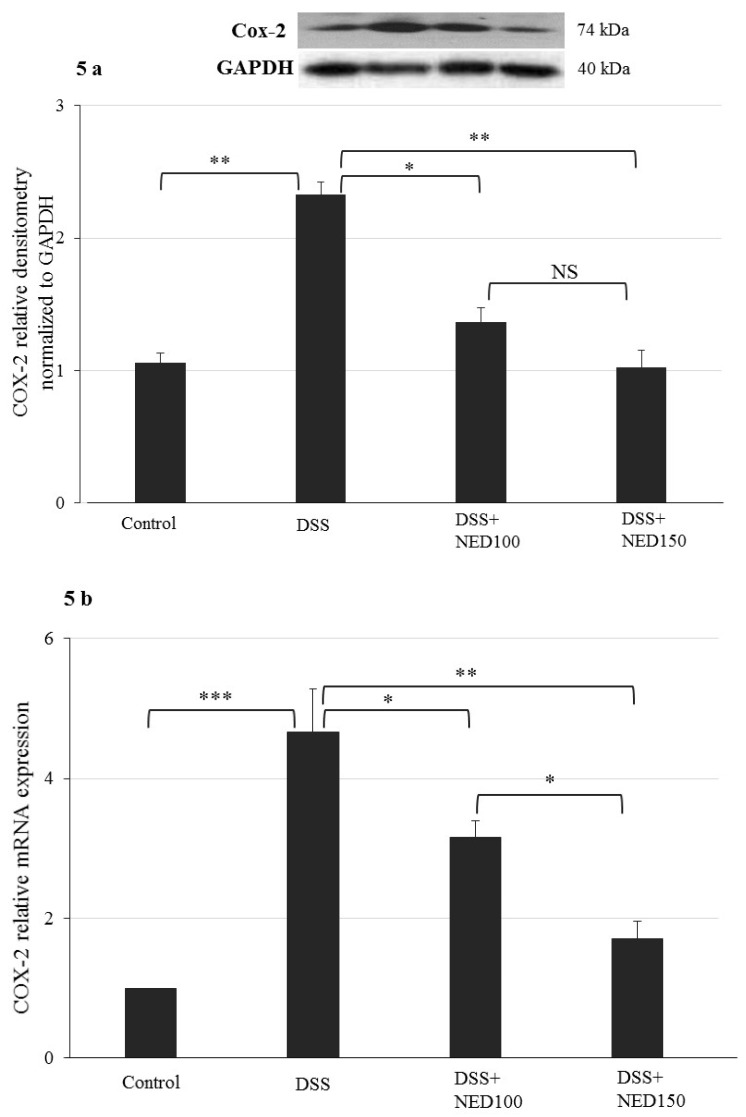

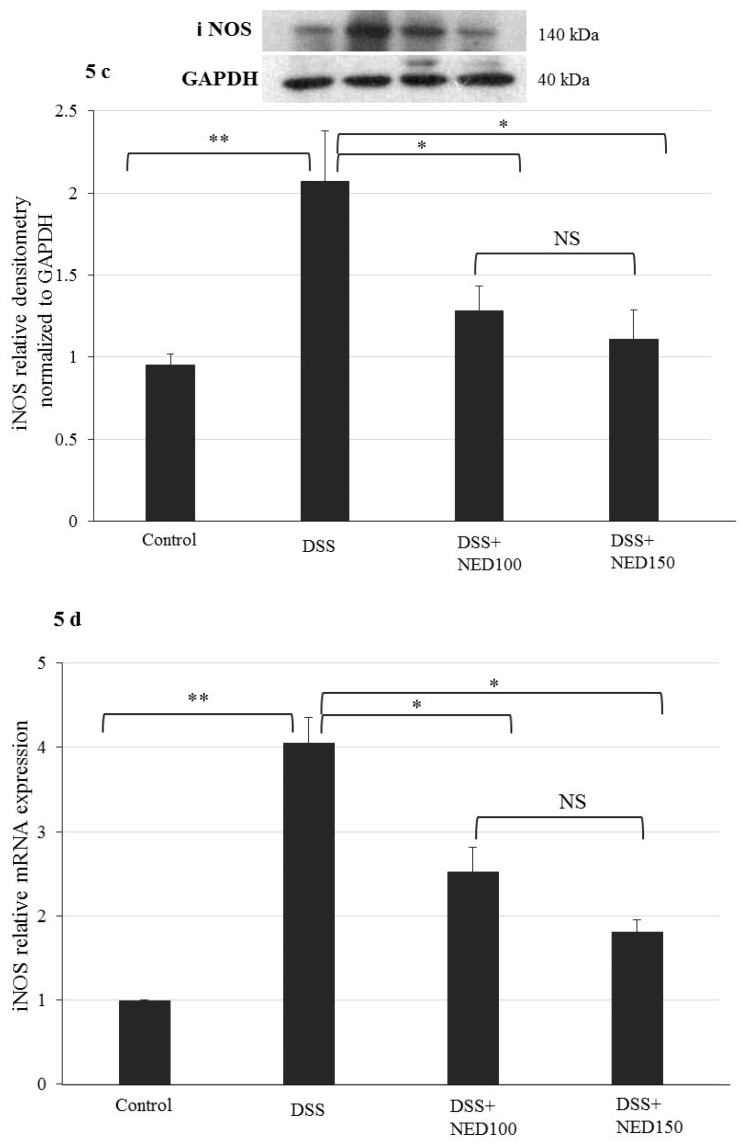

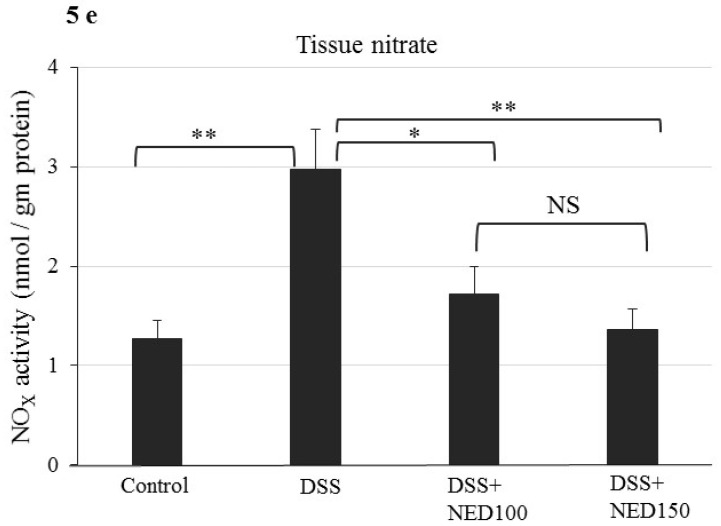
Effect of NED on inflammatory mediator COX-2 and inducible oxide synthase (iNOS) protein and mRNA expression and tissue nitrate levels. NED-treatment significantly inhibited the DSS-induced expression of COX-2 and iNOS at both the protein (**a**,**b**) and mRNA levels (**c**,**d**) and also reduced the tissue nitrate levels (**e**). Data were obtained from *n* = 4 animals for Western blot and *n* = 6 animals for mRNA expression and tissue nitrate level studies in each group and expressed as means ± SEM. *** *p* ≤ 0.001. ** *p* ≤ 0.01. * *p* ≤ 0.05; *p* values were obtained by one-way ANOVA followed by Tukey’s multiple comparison test using SPSS software, and *p* ≤ 0.05 was considered statistically significant.

The effect of NED in DSS treatment was also evaluated on the proinflammatory enzymes COX-2 and iNOS at both the protein and mRNA level. DSS treatment significantly increased COX-2 and iNOS protein and mRNA expression (COX-2; *p* < 0.01; COX-2 mRNA *p* < 0.001; iNOS; *p* < 0.01 and iNOS mRNA *p* < 0.01, Figure 5). NED treatment decreased COX-2 protein and mRNA expression (*p* < 0.05 at 100 mM NED and *p* < 0.01, at 150 mM NED) (Figure 5a,b). Similarly, NED decreased iNOS protein and mRNA expression (*p* < 0.05 at both 100 and 150 mM NED) (Figure 5c,d). A decreased in iNOS both at protein and mRNA level upon NED treatment was correlated with decreased in the tissue nitrate level significantly (*p* < 0.05 at both 100 and 150 mM NED) (Figure 5e).

### 3.4. Effect of NED on Antioxidant Keap-1 and Nrf-2 Transcription Factor Protein, SOD, CAT Activity, and Downstream Target mRNA Expression

**Figure 6:**
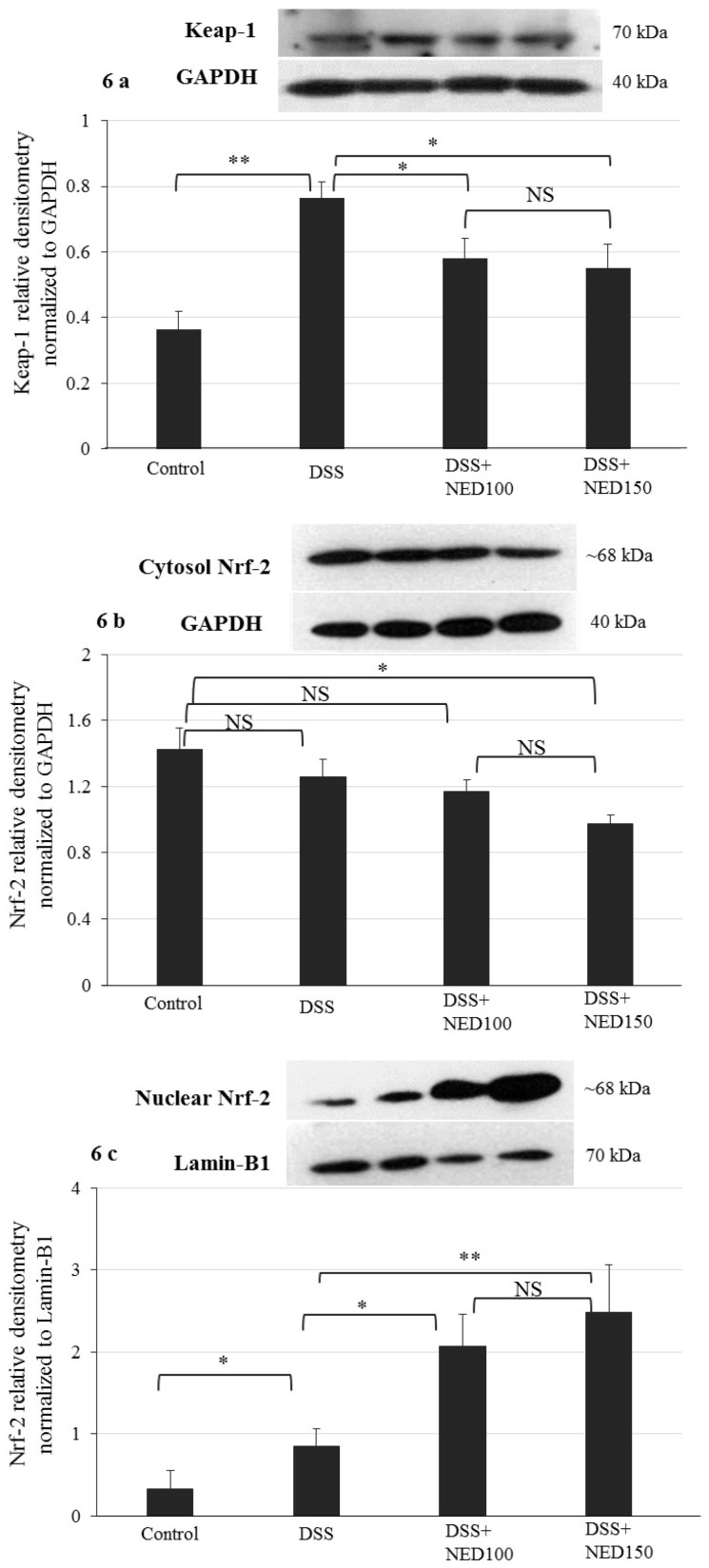

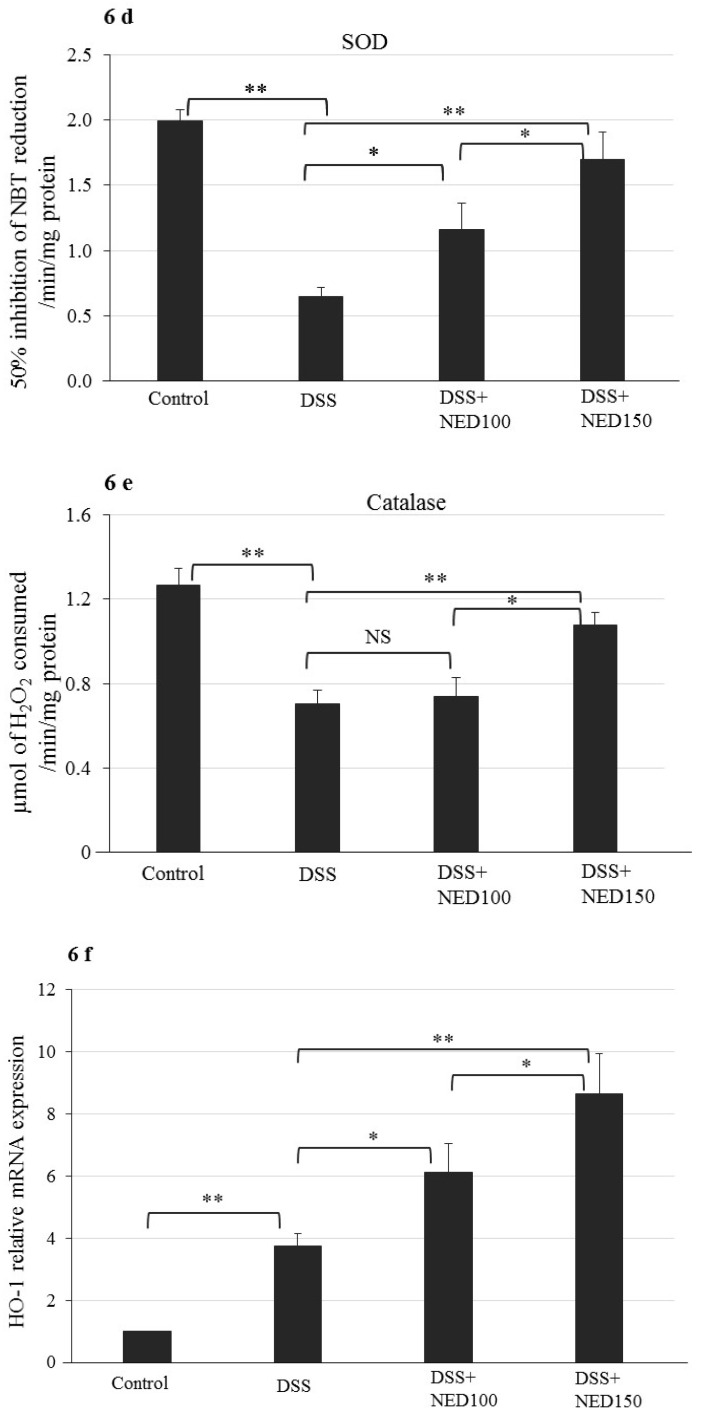

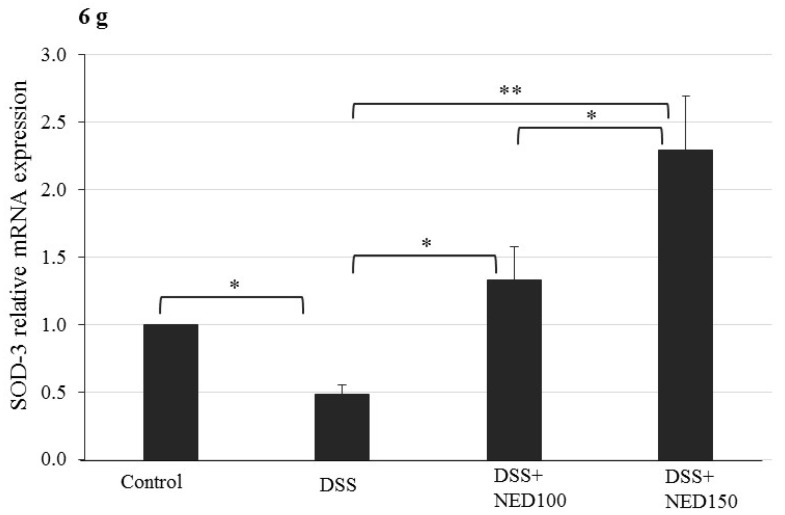
Effect of NED on Keap-1 and Nrf-2 and antioxidant system. At the two higher concentrations, NED prevented the DSS-induced increase in Keap-1 protein expression (**a**). The nuclear translocation of the Nfr-2 protein was enhanced by NED treatment in DSS-induced colitis (**b**,**c**). Superoxide dismutase (SOD) and catalase enzyme activities were significantly increased in NED-treated DSS colitis animals (**d**,**e**). Heme Oxygenase-1 (HO-1) and SOD3 mRNA expression were also significantly increased in NED-treated DSS colitis in mice (**f**,**g**). Data were obtained from *n* = 4 animals for the Western blot studies, *n* = 6 for enzyme activity and mRNA level. Data expressed as means ± SEM. *** *p* ≤ 0.001. ** *p* ≤ 0.01. * *p* ≤ 0.05. *p* values were obtained by one-way ANOVA followed by Tukey’s multiple comparison test using SPSS software, and *p* ≤ 0.05 was considered statistically significant.

Nuclear factor-erythroid 2-related factor (Nrf-2) is a transcription factor of great significance because it is the major regulator of the expression of genes codes for antioxidant enzymes. Keap-1 is a repressor that binds to Nrf-2 and promotes its degradation via the ubiquitin proteasome pathway. We investigated the effect of NED on the Keap-1 and Nrf-2 system. DSS treatment increased Keap-1 expression in the cytoplasm, and NED treatment significantly decreased this response (*p* < 0.05) (Figure 6a). Under basal conditions, Keap-1 binds to Nrf-2 in the cytoplasm; however, upon activation, Nrf-2 translocates into the nucleus and binds to the antioxidant response element. Therefore, Nrf-2 expression carried out using both cytoplasm and the nuclear fraction of colonic epithelial cells. In the cytoplasm, DSS treatment did not affect the Nrf-2 protein level compared to control; however, the high dose of NED treatment significantly (*p <* 0.05) decreased the Nrf-2 level (Figure 6b). In contrast, the concentration of Nrf-2 in the nuclear fraction was modestly increased by DSS treatment (*p <* 0.05) and was statistically increased in a concentration-dependent manner by NED (*p <* 0.05 at 100 µM and *p <* 0.01 at 150 µM) (Figure 6c). Numerous cytoprotective genes are regulated by Nrf-2 when translocated into the nucleus [35]. Therefore, tissue superoxide dismutase (SOD) and catalase enzyme activity was determined in NED-treated DSS colitis animals along with expression of Heme Oxygenase-1 (HO-1) and SOD3 mRNA. NED treatment significantly increased colon mucosal SOD (*p <* 0.05 at 100 mM and *p <* 0.01 at 150 mM) activity (Figure 6e). However, the catalase activity was increased only at higher dose of NED in statistically significant manner (*p <* 0.01 at 150 µM) (Figure 6f). NED treatment also increased HO-1 mRNA (*p <* 0.05 at 100 mM and *p <* 0.01 at 150 mM) (Figure 6g) and SOD3 mRNA expression significantly (*p <* 0.05 at 100 mM and *p <* 0.01 at 150 mM) in DSS colitis colon (Figure 6g).

### 3.5. Effect of NED on TNF-α stimulated HT-29 Colon Cells

**Figure 7:**
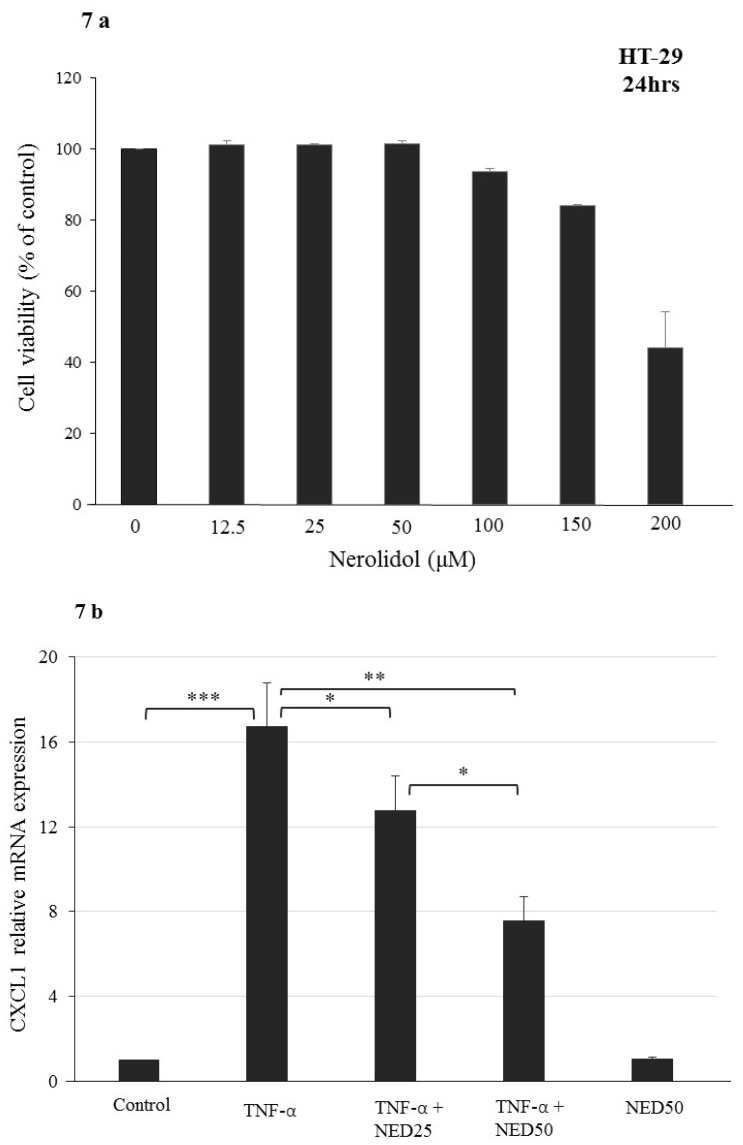

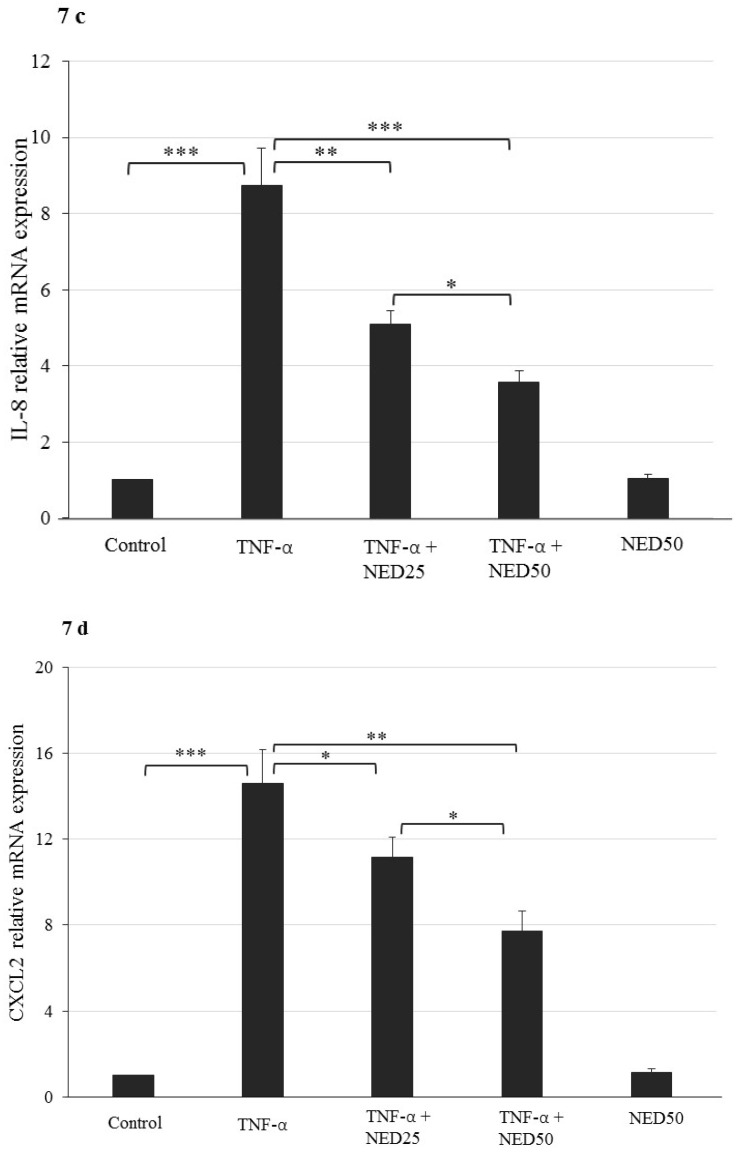

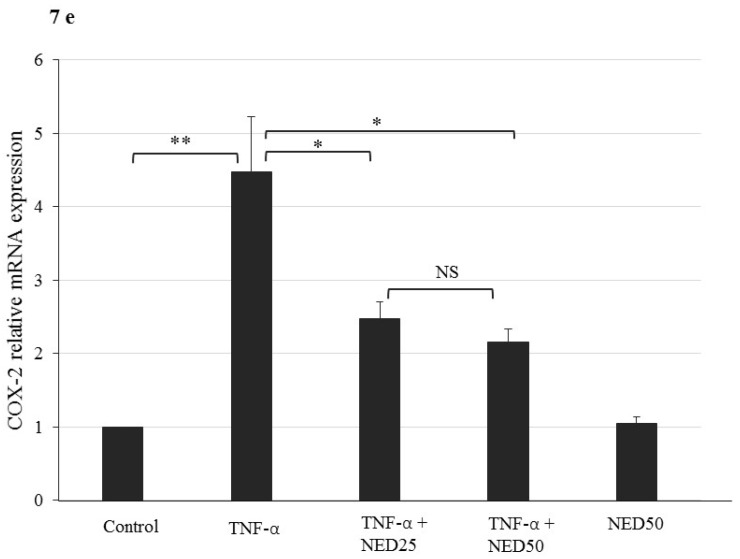
Effect of NED on cell viability and proinflammatory cytokine mRNA expression in TNF-α-treated HT-29 human colonic cancer cells. The concentration-dependent cytotoxic effects of NED were investigated treatment of HT-29 cells with different concentrations of NED for 24 h. NED had no effect on HT-29 cell viability at concentrations up to 100 µM, so its anti-inflammatory effects were examined at concentrations of 25 and 50 µM in the subsequent experiments (**a**). To investigate the effect of NED on inflammatory cytokine production, cells were treated with 1 ng/mL TNF-α for 24 h. Real-time PCR was carried out to detect gene expression of the inflammatory markers CXCl1, CXCL2, IL-8, and COX-2 (**b**–**e**). Data are shown relative to DMSO vehicle-treated cells. Data expressed as mean ± SEM (*n* = 6). *** *p* ≤ 0.001. ** *p* ≤ 0.01. * *p* ≤ 0.05. *p* values were obtained by one-way ANOVA followed by Tukey’s multiple comparison test using SPSS software. *p* ≤ 0.05 was considered statistically significant, and NS indicates not significant.

We evaluated the anti-inflammatory effect of NED in a disease-relevant cellular context by using TNF-α stimulated HT-29 colon cancer cells in culture, a well-established model for inflammatory process in the gut [34]. To eliminate the possibility of cytotoxicity of NED in HT-29 colon cells and to optimize treatment, we treated these cells with different concentrations (in micromolar range) of NED for 24 hr. No cytotoxic effect observed up to 150 µM concentration. Only at 200 µM, a slight but significant (*p* < 0.05) cytotoxic effect was observed (Figure 7a). Therefore, the subsequent experiments carried out using 25 to 50 µM NED, concentrations well within the non-cytotoxic range. HT-29 cells stimulated with TNF-α showed a significant increase in the mRNA of the inflammatory marker genes, CXCL1, CXCL2, IL-8 (*p* < 0.001), and COX-2 (*p* < 0.01). All these proinflammatory gene markers were significantly downregulated upon NED treatment in TNF-α stimulated HT-29 cells (Figure 7b–e). However, NED alone did not induce any change in gene expression profile compared to the control treatment. These results further confirm the potent anti-inflammatory properties of NED.

## 4. Discussion

In the present study, we investigated the role of NED in mitigating the colonic inflammation induced by DSS in mice; a well-accepted experimental model of ulcerative colitis. Oral administration of NED alleviated DSS-induced weight loss as well as clinical symptoms as reflected by the DAI score in C56B7/6J mice at a doses of 100 and 150 mg/kg/day PO, which were confirmed by macroscopic, histological and biochemical examinations. High DAI scores and a shortened colon represent the pathological state and the severity of colon in the colitis model group. At the two higher concentrations (100 and 150 mg/kg/day) NED was more effective than the control drug, sulfasalazine (50 mg/kg/day) in reducing inflammation as indicated by all of the studies undertaken. However, the lowest dose of NED (50 m/kg/day) had no significant effect on any inflammatory parameter.

NED significantly prevented the DSS-induced reduction of colon length, and a dose-dependent reduction in the DAI score was also observed with NED treatment. The DSS colitis mice colons exhibited marked histopathological changes, including epithelial erosion and ulceration, crypt abscess formation, mucosal erosion and edema, and substantial infiltration of macrophages. Although no significant effect was observed at 50 mg/kg dose, NED administration was able to preserve near to normal microscopic architecture of colon at the higher doses. Colonic MPO was increased in DSS colitis mice statistically. Upregulated MPO leads to weakened antioxidant defense mechanisms, which will facilitate oxidative injury [36]. NED remarkably reduced inflammation-induced neutrophil infiltration. CXCL2 and CCL2 are known as neutrophil and macrophage chemoattractant chemokines involved in immune responses [37,38]. The DSS-induced increase in mRNA levels of these chemoattractants were significantly prevented by NED.

Infiltration of neutrophils into colonic tissue causes mucosal damage by releasing reactive oxygen species (ROS), which eventually results in the induction of proinflammatory cytokine release, especially TNF-α [4,28]. TNF-α is a primary mediator of the inflammatory response and is closely linked to colonic inflammation of UC [29]. Excessive cytokine production due to immune response is implicated in the intestinal inflammation and the extraintestinal manifestations in IBD [39]. We observed that the elevated tissue levels of IL-6, IL-1β, and TNF-α in DSS treated animals were significantly reduced by higher doses (100 mg and 150 mg/kg bd wt) of NED treatment. These findings are compatible with previous reports, where NED was shown to decrease the influx of polymorphonuclear cells in carrageenan-induced peritonitis, reduce levels of TNF-α in the peritoneal lavage, and reduce production of IL-1β in LPS-stimulated, peritoneal macrophages [27]. These observations indicate that this sesquiterpene has potent anti-inflammatory properties.

Proinflammatory cytokines at the site of inflammation induce cyclooxygenase 2 (COX-2) expression which results in the increased synthesis of prostaglandins and amplification of mucosal inflammation in the intestinal epithelium of IBD patients [40]. Inflammatory cytokines, such as IL-6, are known to upregulate COX-2 expression [41]. Our results reveal increased expression of COX-2 at both the mRNA and protein levels in DSS-treated animals. This increase in COX-2 expression was significantly reduced by NED treatment. In a previous study, NED was shown to suppress TNF-α and IL-6 [42]; therefore, the observed inhibition of COX-2 activity may be facilitated through the suppression of these cytokines. Inducible nitric oxide synthase (iNOS) is expressed in response to bacterial/proinflammatory stimuli and results in nitric oxide (NO) production, which provides cytoprotection. Excessive iNOS expression leads the upregulated NO turnover that is associated with the pathogenesis of IBD [43]. The up-regulation of inducible oxide synthase (iNOS) expression is induced by proinflammatory cytokines in IBD [44]. Our results show that iNOS, a macrophage activation maker, was increased in colonic tissues consequent to DSS-induced injury, as demonstrated by quantitative RT-PCR and Western blotting. NED suppressed the expression of COX-2 and iNOS at both the mRNA and protein levels significantly. In lipopolysaccharide (LPS)-stimulated RAW macrophages model of inflammation, the essential oil from *Lindera erythrocarpa*, which contains NED, inhibits the expression of iNOS and COX-2 and decreases the subsequent production of NO and prostaglandin E_2_, respectively [45]. Similarly, NED inhibited iNOS and COX-2 protein expression in a rat model of neuro-inflammation [28]. To address the role of iNOS-derived NO in colitis, studies have been carried out using various iNOS specific inhibitors in in vivo colitis models. However, the results emanating from these studies have revealed conflicting results [46].

Under physiological conditions, a dimer of Nrf2 and Keap1 exists in the cytoplasm. In response to electrophiles or ROS, Nrf2 dissociates from Keap1, which is a negative modulator of Nrf2, and this results in translocation of Nrf2 into the nucleus, where it activates transcription of numerous genes coding for the enzymes involved in antioxidant defense mechanisms [47]. Our results indicate that NED promotes the nuclear translocation of Nrf2 and a decrease in the expression of Keap-1. These results are in agreement with previous studies where sesquiterpenes activate Nrf2 nuclear translocation to modulate antioxidant enzyme expression [48,49,50]. The mRNA expression of heme oxygenase-1 (HO-1) and superoxide dismutase-3 (SOD3), which are among the downstream targets of Nrf-2, were also analyzed. HO-1 is an important enzyme for catabolism of several heme-containing proteins including hemoglobin, myoglobin, and cytochrome p450. A basal level of expression is seen under physiological conditions, but HO-1 is highly expressed in response to stressful stimuli [35,51]. This increased HO-1 expression offers protection during intestinal inflammation [52,53]. Dose-dependent increases in expression of HO-1 mRNA observed in the NED-treated group may provide protection from inflammation. In the present study, SOD3 mRNA expression was decreased in the DSS group, but dose-dependently increased by NED treatment to levels higher than control group. Among the three isoforms of SOD, SOD-3 was shown to be decreased in intestinal epithelial cells (IECs) [54]. It is also reported that increased SOD3 mRNA expression resulted in apoptosis and death of cancer cells and suggestive of cell viability [55]. In a rat model of rotenone-induced Parkinson’s disease, NED maintained cellular SOD, CAT, and GSH and decreased the level of MDA significantly [28]. These results are in agreement with our findings.

The anti-inflammatory activity of NED was also examined using TNF-α-induced HT-29 cells, which are human colonic adenocarcinoma cells with an epithelial morphology. The effects of NED on the expression of COX-2, CXCL1, CXCL2, and IL8 mRNA were measured in cells exposed to TNF-α to induce an inflammatory state. Chemokine ligand-2 (CXCL2) is a chemotactic chemokine produced by colonic epithelial cells and macrophages in response to infection or injury [56]; IL8 is the most active neutrophil chemoattractant that is upregulated during inflammation [34,57]. Chemokine ligand-1 (CXCL1) plays a role in inflammation and tumorigenesis [58]. In colonic mucosa of IBD patients, increased IL-8 and CXCL1 are positively correlated with the disease activity [59]. The results of our study in TNF-α-treated HT-29 cells confirm that NED has a potent anti-inflammatory property. The reductions seen in mRNA expression may be partly attributed to the observations from a study where NED containing essential oil from *Liquidambar formosana* leaves the inflammatory response suppressed in LPS-induced macrophages [60].

## 5. Conclusions

NED, which has been shown to suppress inflammation in several different models, exhibited statistically significant anti-inflammatory effects in a well-studied model of inflammatory bowel disease. It was notable that the effects of this compound were at least as efficacious as those of sulfasalazine, a drug commonly used in treatment of Crohn’s disease and ulcerative colitis. Safety appears not to be an issue because NED is used widely as a flavor enhancer in the food industry and has approval from the US Food and Drug Administration for use in food. NED or a synthetic derivative may be valuable in the treatment of inflammatory bowel disease and perhaps other inflammatory conditions too.

## Figures and Tables

**Table 1:** Disease activity index score.

Weight Loss	Score	Stool Consistency	Score	Rectal Bleeding	Score
No loss	0	Normal	0	No Blood	0
1–5%	1	Loose stool	2	Heme occult + ve and visual pellet bleeding	2
5–10%	2	Diarrhea	4	Gross bleeding and blood around anus	4
10–20%	3				
>20%	4				

**Table 2:** Colon tissue histological examination Crypt aberration score.

Inflammation Graded	Percentage of Inflammation Involvement of Mucosal Surface Area		Hyperplastic Epithelium—Graded Based on Extent of Involvement	
none	0	no inflammation	0	none
mild	1	1–25%	1	1–25%
moderate	2	26–50%	2	26–50%
severe	3	51–75%	3	51–75%
	4	76–100%	4	76–100%

**Table 3:** Colon tissue histological examination for inflammation Score.

Crypt Grade		Quantification on Percentage of Crypt Change		Crypt Distortion—Graded Based on Extent of Involvement	
Grade 0	Intact crypt	1	1–25%	0	no crypt distortion
Grade 1	Shortening and loss of basal 1/3 of crypts	2	26–50%	1	1–25%
Grade 2	Loss of basal 2/3 of crypts	3	51–75%	2	26–50%
Grade 3	Loss of entire crypt with intact surface epithelium	4	76–100%	3	51–75%
Grade 4	Loss of both entire crypt and surface epithelium (erosion)			4	76–100%

**Table 4:** Primers list.

Gene	Forward	Reverse	PMID No	Gene Accession Number
CXCL2 (mouse)	5′-GGATGGCTTTCATGGAAGGAG-3′	5′-TTGCTAAGCAAGGCACTGTGC-3′	22326488	NM_009140.2
CCL2 (mouse)	5′-CAGCCAGATGCAGTTAACGC-3′	5′-GCCTACTCATTGGGATCATCTTG-3′	10953027	NM_011333.2
IL-6 (mouse)	5′-ACAAGTCGGAGGCTTAATTACACAT-3′	5′-TTGCCATTGCACAACTCTTTTC-3′	21735552	X06203
TNF-α (mouse)	5′-AGGCTGCCCCGACTACGT-3′	5′-GACTTTCTCCTGGTATGAGATAGCAAA-3′	21705622	NM_013693.2
IL-1β (mouse)	5′-TCGCTCAGGGTCACAAGAAA-3′	5′-CATCAGAGGCAAGGAGGAAAC-3′	21735552	NM_008361.4
COX-2 (mouse)	5′-AACCGCATTGCCTCTGAAT-3′	5′-CATGTTCCAGGAGGATGGAG-3′	22158945	NM_011198.4
iNOS (mouse)	5′-CGAAACGCTTCACTTCCAA-3′	5′-TGAGCCTATATTGCTGTGGCT-3′	22158945	BC062378.1
NRF-2 (mouse)	5′-GAGCTAGATAGTGCCCCTGG-3′	5′-CAGGACTCACGGGAACTTCT-3′	29162986	U20532.1
HO-1 (mouse)	5′-AAGCCGAGAATGCTGAGTTCA-3′	5′-GCCGTGTAGATATGGTACAAGGA-3′	25112868	BC010757.1
SOD3 (mouse)	5′-TTCTACGGCTTGCTACTGGC-3′	5′-GCTAGGTCGAAGCTGGACTC-3′	26513461	NM_011435.3
18S (mouse)	5′-CCCCTCGATGACTTTAGCTGAGTGT-3′	5′-CGCCGGTCCAAGAATTTCACCTCT-3′	22427817	NR_003278
CXCL1 (human)	5′-GCGGAAAGCTTGCCTCAATC-3′	5′-GGTCAGTTGGATTTGTCACTGT-3′	25938459	BC011976.1
IL-8 (human)	5′-CTGATTTCTGCAGCTCTGTG-3′	5′-GGGTGGAAAGGTTTGGAGTATG-3′	20150959	BC013615.1
CXCL2 (human)	5′-TTTATTGTGGGCTTCACACG-3′	5′-GATTTGCGCACACAGACAAC-3′	17591792	NM_004591.3
COX2 (human)	5′-ACAGTGTGTGGTCAACATTTCTC-3′	5′-TCGAAACCTCTCTGCTCTAACAC-3′	25938459	BC015753.1
18S (human)	5′-GTGGAGCGATTTGTCTGGTT-3′	5′-AACGCCACTTGTCCCTCTAA-3′	25369870	NR_003286
